# Effect and mechanism of *Plantaginis Semen polysaccharides* on intestinal microecology in rats with hyperuricemia

**DOI:** 10.3389/fmicb.2025.1555734

**Published:** 2025-03-26

**Authors:** Jing Zhao, Yu Fu, Hongbin Qiu

**Affiliations:** ^1^Key Laboratory of Microecology-immune Regulatory Network and Related Diseases School of Basic Medicine, Jiamusi University, Jiamusi, China; ^2^Department of Medical Laboratory, The First Affiliated Hospital of Jiamusi University, Jiamusi, China; ^3^Laboratory Department, Center for Disease Control and Prevention of Jiamusi, Jiamusi, China

**Keywords:** hyperuricemia, *Plantaginis Semen polysaccharides*, intestinal microecology, uric acid, kidney

## Abstract

**Introduction:**

Hyperuricemia (HUA) is characterized by metabolic abnormalities in purine metabolism, leading to an excessive accumulation of uric acid in the bloodstream. This condition is not only prevalent but also significantly linked to the exacerbation of various chronic diseases, including renal impairment. Notably, asymptomatic hyperuricemia is frequently associated with inflammatory responses and alterations in intestinal microbiota. Thus, it is imperative to explore effective therapeutic interventions for HUA to mitigate its associated health risks.

**Methods:**

The present study aimed to elucidate the protective effects of *Plantaginis Semen polysaccharides* (PSP) in a rat model of hyperuricemia induced by adenine (AD) and potassium oxonate (PO) gavage. Over a treatment period of five weeks, the animals received either PSP or allopurinol (AL). Comprehensive assessments were performed, including blood biochemistry analysis, histopathological evaluation, Western blot analyses to investigate the expression levels of key renal transport proteins, as well as 16S rRNA sequencing to explore microbiota shifts.

**Results:**

The findings demonstrated that PSP significantly decreased serum uric acid (UA) levels and alleviated renal dysfunction through modulation of xanthine oxidase (XOD) and adenosine deaminase (ADA) serum concentrations and the expression of renal transporters, namely glucose transporter protein 9 (GLUT9), urate transporter 1 (URAT1), ATP-binding cassette superfamily member 2 (ABCG2), and organic anion transporter 1 (OAT1). Furthermore, PSP exhibited notable anti-inflammatory properties, reflected in the reduced levels of pro-inflammatory cytokines such as tumor necrosis factor-α (TNF-α) and interleukin-1β (IL-1β).

**Conclusion:**

In summary, the present study substantiates the therapeutic potential of PSP in treating hyperuricemia through its dual action of lowering uric acid levels and imparting anti-inflammatory effects. The observed modulation of gut microbiota further supports the role of PSP in maintaining metabolic homeostasis. Future investigations should focus on the clinical applicability of PSP and elucidate the mechanisms underlying its beneficial impacts on hyperuricemia and associated metabolic disorders.

## 1 Introduction

Hyperuricemia (HUA) is a chronic metabolic disease caused by purine metabolism disorders and has become the fourth major metabolic abnormality following hyperlipidemia, hyperglycemia, and hypertension, with its incidence continuously rising and posing significant public health challenges (Choi et al., 2020; Dalbeth et al., 2021). The pathological features of HUA progressing from an asymptomatic state to gouty arthritis and renal failure are becoming increasingly clear (Wang et al., 2022). As the end product of purine metabolism, the homeostasis of uric acid in the body relies on liver and kidney regulation; excessive accumulation can induce oxidative stress, systemic inflammation, and organ damage (Tasic et al., 2016; Hu et al., 2021). In recent years, gut microbiota has been confirmed to profoundly influence UA homeostasis through metabolic regulation and immune cross-talk: specific strains can directly degrade uric acid or regulate host purine metabolic pathways (Li et al., 2019; Sakurai et al., 2022).

Gut microbiota play a key role in maintaining intestinal barrier integrity, immune balance, and nutritional metabolism, and their ecological imbalance (such as a reduction in *bifidobacteria*/*lactobacilli*) is closely related to metabolic syndrome (Gerritsen et al., 2011; Adak and Khan, 2019). Notably, about 25% of UA is excreted through the intestine and is regulated by microbial metabolism (Shi et al., 2023). HUA patients exhibit characteristic dysbiosis (Chen et al., 2021b; Zhou et al., 2021), and a high-purine diet or pathological stress can exacerbate microbial dysregulation, disrupt tight junction proteins leading to intestinal barrier leakage, promote the entry of microbial metabolites into the bloodstream, and activate the NLRP3 inflammasome, ultimately resulting in kidney damage (Lin et al., 2021). This “microbiota-metabolism-inflammation” vicious cycle suggests that targeting gut microbiota regulation may be a novel strategy for intervening in HUA.

The most frequently prescribed UA-lowering medications in clinics include benzbromarone, allopurinol, and febuxostat. These drugs have demonstrated rapid and significant therapeutic effects against HUA. However, prolonged use of these medications can lead to serious side effects that should not be overlooked. For example, the long-term use of allopurinol is closely associated with the risk of severe skin damage, and the incidence of this adverse effect exceeds 10%, which may even be life-threatening in extreme cases. Consequently, investigating natural active ingredients derived from plants that can effectively lower UA while ensuring high safety is paramount. Natural plant-based medicine offers unique benefits in HUA prevention and treatment, thanks to its mild action and low toxicity, making it a key focus in food science and pharmaceutical research. Prior research has indicated that specific plant extracts exert positive effects on UA metabolism and renal protection, and its natural source characteristics and low toxicity provide a safer metabolic regulation strategy for the treatment of disease. This also provides a theoretical basis for this study thereby establishing a theoretical foundation for the present study (Koseki et al., 2022).

*Plantaginis Semen polysaccharides* (PSPs) are natural polysaccharides extracted from *Plantago asiatica* (*P. asiatica*) seeds. PSPs are the main active ingredients in *P. asiatica*. Research has shown that PSPs play crucial roles in immune regulation and enhance resistance (Khedher et al., 2022). Adjuvant therapy has significant positive effects on chronic inflammatory diseases. PSPs have antioxidant properties that help eliminate free radicals in the body, thus decelerating the aging process and safeguarding cells from oxidative damage (Zhang et al., 2021). Simultaneously, PSPs demonstrate the ability to reduce blood sugar and blood lipid levels, highlighting their promising applications in health care food and medicine (Fan et al., 2021). Previous studies have shown that PSPs can effectively reduce serum UA levels *in vitro* (Zhao et al., 2021). However, there is a paucity of literature exploring the mechanism by which PSPs reduce UA from kidney function and gut microbiota perspectives.

Consequently, the present study explored the effect and underlying mechanisms of PSPs on intestinal microecology in a rat model of HUA. This study seeks to provide a basis for developing new, effective, and low-toxicity drugs for HUA treatment.

## 2 Materials and methods

### 2.1 Reagents and materials

*Plantago asiatica* was purchased from a drugstore in Wuchang City, Heilongjiang Province, China. Adenine and potassium oxonate were sourced from Shanghai Yuanye Biotechnology Co., Ltd. (Shanghai, China). Allopurinol tablets were sourced from Shanghai Xinyi Wanxiang Pharmaceutical Co., Ltd. (Shanghai, China). The enzyme-linked immunosorbent assay (ELISA) kits for measuring xanthine oxidase (XOD), tumor necrosis factor-alpha (TNF-α), interleukin-1 beta (IL-1β), diamine oxidase (DAO), and lipopolysaccharide (LPS) in rats were obtained from Jiangsu Meimian Biotech Co., Ltd. (Jiangsu, China). Reagents for assessing serum (SUA), serum creatinine (SCR), blood urea nitrogen (BUN), and serum adenosine deaminase (ADA) were supplied by Beckman Coulter, Inc. (Florida, United States). All antibodies utilized in this study were provided by Wuhan Sanying Biotechnology Co., Ltd. (Hubei, China). Additionally, bicinchoninic acid (BCA) protein concentration assay reagents were procured from Beyotime (Shanghai, China).

### 2.2 Extraction of crude polysaccharides from *Plantaginis Semen*

*Plantaginis Semen* was extracted with 10 times the volume of distilled water (100°C, 2 h, repeated thrice). The integrated three aqueous extracts were collected and concentrated. The concentrate was added to 95% ethanol and stirred uniformly to a final concentration of 83%. The alcohol was precipitated overnight, and the precipitate was obtained by centrifugation. The precipitate was re-dissolved and volatilized in ethanol. The obtained extract was deproteinated using the Sevage method and freeze-dried to obtain plantain polysaccharide.

### 2.3 Establishment of a rat model of HUA and drug administration

A rat model of HUA was established to evaluate the effect of PSP on HUA as previously described (Chen et al., 2022; Zhang et al., 2022). Male Sprague Dawley rats (180–200 g) were purchased from the Department of Experimental Zoology of Harbin Medical University. The animals were raised in the animal room under controlled conditions (temperature: 22–24°C, relative humidity: 50–60%, and a 12/12-h day/dark cycle), with free access to food and water. All animal experimental procedures in this study strictly adhered to the international Guidelines for the Care and Use of Laboratory Animals. The experimental protocol was reviewed and approved by the Animal Experimental Ethics Committee of Jiamusi University (Approval No.: 202245). All rats were acclimated for 1 week before the experiment and then randomly divided into six groups (*n* = 10 rats/group): blank control group (control), model group (Model), positive drug control group, i.e., allopurinol group (AL), low-dose PSP group (PSP_L), medium-dose PSP group (PSP_M), and high-dose PSP group (PSP_H). All rats, except for those in the control group, were gavaged with 100 mg/kg of adenine +300 mg/kg potassium oxonate mixed in 0.5% sodium carboxymethyl cellulose once daily to establish the hyperuricemic rat model, and the rats were fed food mixed with yeast. The control group received 0.5% carboxymethyl cellulose sodium (CMC-Na) by gavage. Treatment was increased on day 8, where control and model groups were gavaged with equal amounts of saline. AL, PSP_L, PSP_M, and PSP_H groups were gavaged with 5 mg/kg, 1.35 g/kg, 2.7 g/kg, and 5.4 g/kg, respectively. The experiment lasted for 7 weeks, and the body weight was recorded daily.

After the experiment, blood, kidneys, intestines, and intestinal contents were collected 1 h after the last administration. Blood specimens were centrifuged at 3,500 rpm for 15 min to obtain serum, which was subsequently preserved at −80°C in a freezer until analysis. The right kidney tissues from each rat were fixed in a 4% paraformaldehyde solution and then embedded in paraffin for histopathological examination. Additionally, residual tissues and intestinal contents were rapidly frozen at −80°C in liquid nitrogen for subsequent analyses.

### 2.4 Histopathological analysis

Pre-fixed kidney tissues that were thoroughly rinsed, dehydrated, and then embedded in paraffin. Subsequently, the samples were cut into 4 μm thick slices and then stained with hematoxylin and eosin (HE). Morphological alterations were examined under a microscope.

### 2.5 Analysis of biochemical indexes, inflammatory factors, and renal index

SUA, CR, BUN, and ADA levels were detected using an automatic biochemical analyzer (Beckman au5800, California, United States). TNF-α, IL-1β, XOD, LPS, and DAO concentrations were measured utilizing commercially available ELISA kits, following the manufacturer’s protocols. The renal index was expressed as double kidney weight/body weight × 100%.

### 2.6 Western blot analysis

Proteins were extracted from kidney tissues utilizing the radioimmunoprecipitation assay (RIPA) Protein Lysis Buffer (Beyotime, Shanghai, China). Protein concentration was quantified employing the BCA Protein Assay Kit. Afterward, proteins were separated through 10% sodium dodecyl sulfate-polyacrylamide gel electrophoresis (SDS-PAGE) and then transferred to polyvinylidene difluoride (PVDF) membranes. Membranes were blocked utilizing 5% skim milk at room temperature for 90 min. After discarding the sealing fluid, membranes were incubated at 4°C overnight with specific primary antibodies: ATP binding cassette subfamily G member 2 (ABCG2), organic anion transporter 1 (OAT1), glucose transporter 9 (GLUT9), urate transporter 1 (URAT1), zonula occludens-1 (ZO-1), nucleotide-binding domain, leucine-rich-containing family, pyrin domain-containing-3 (NLRP3), TNF-α, occludin, caspase-1, and IL-1β (Sanying, Hubei, China). After rinsing with phosphate-buffered saline, membranes were incubated with a horseradish peroxidase (HRP) -conjugated secondary antibody at room temperature for 1 h. Blots were developed and exposed with an enhanced chemiluminescence (ECL) detection kit. The integral optical density was utilized to assess the ratio of the gray value of the target protein relative to that of the reference protein.

### 2.7 16S rRNA sequencing analysis

To explore the relationship between the composition of intestinal flora and HUA rats, three intestinal contents were selected from each group for 16S rRNA analysis. Genomic DNA was extracted from intestinal contents using magpure soil DNA LQ Kit (Magan, China). Qualified DNA samples and corresponding fusion primers were employed to configure the PCR reaction system for PCR amplification. The amplified products were purified and used to construct the library. Libraries were assayed using an Agilent 2100 Bioanalyzer, and the HiSeq platform was used to perform the sequencing. Lower machine data filtering, stitching reads into tags based on the overlap relationship and sequence stitching were performed using FLASH software. The spliced tags were clustered using the USEARCH software (v7.0.1090) into operational taxonomic units (OTUs) and analyze the microbial diversity and community composition structure based on the OTUs.

Species notes: Venn diagrams were utilized to visualize shared operational taxonomic units (OTUs) among groups and quantify unique OTUs within each group. Alpha Diversity Assessment: Species accumulation curves were generated to comparatively evaluate microbial richness (Chao1 index) and community evenness (Shannon/Simpson indices) across experimental groups. Beta Diversity Profiling: Principal coordinate analysis (PCoA) was implemented to elucidate structural variations in microbial communities between samples using Bray–Curtis dissimilarity matrices. Signature Microbiota Characterization: Taxonomic composition tables at multiple classification levels (phylum/genus) were derived through QIIME2 pipeline analysis, with differential taxa visualized via hierarchical clustering heatmaps. Linear discriminant analysis effect size (LEfSe) was employed (LDA score > 2.0, *p* < 0.05) to identify phylogenetically discriminative features. Metabolic Pathway Enrichment: Pathway-centric analysis was conducted to capture global flux alterations across metabolic networks. The top 10 significantly enriched pathways (Benjamini-Hochberg adjusted *p* < 0.05) underwent differential abundance scoring using weighted Z-score transformations to map systemic pathway perturbations (Yang et al., 2024).

### 2.8 Statistical analysis

All statistical analyses were conducted employing the software programs Prism 8.0 (GraphPad Software Inc., San Diego, CA, United States) and SPSS 25.0 (SPSS Inc., Chicago, United States). The results were expressed as means ± standard deviations (SD) and statistical differences were compared using one-way analysis of variance (ANOVA), followed by Dunnett’s test. *p* < 0.05 was considered statically significant.

## 3 Results

### 3.1 PSP reduces the level of SUA in HUA rats

**Figure 1:**
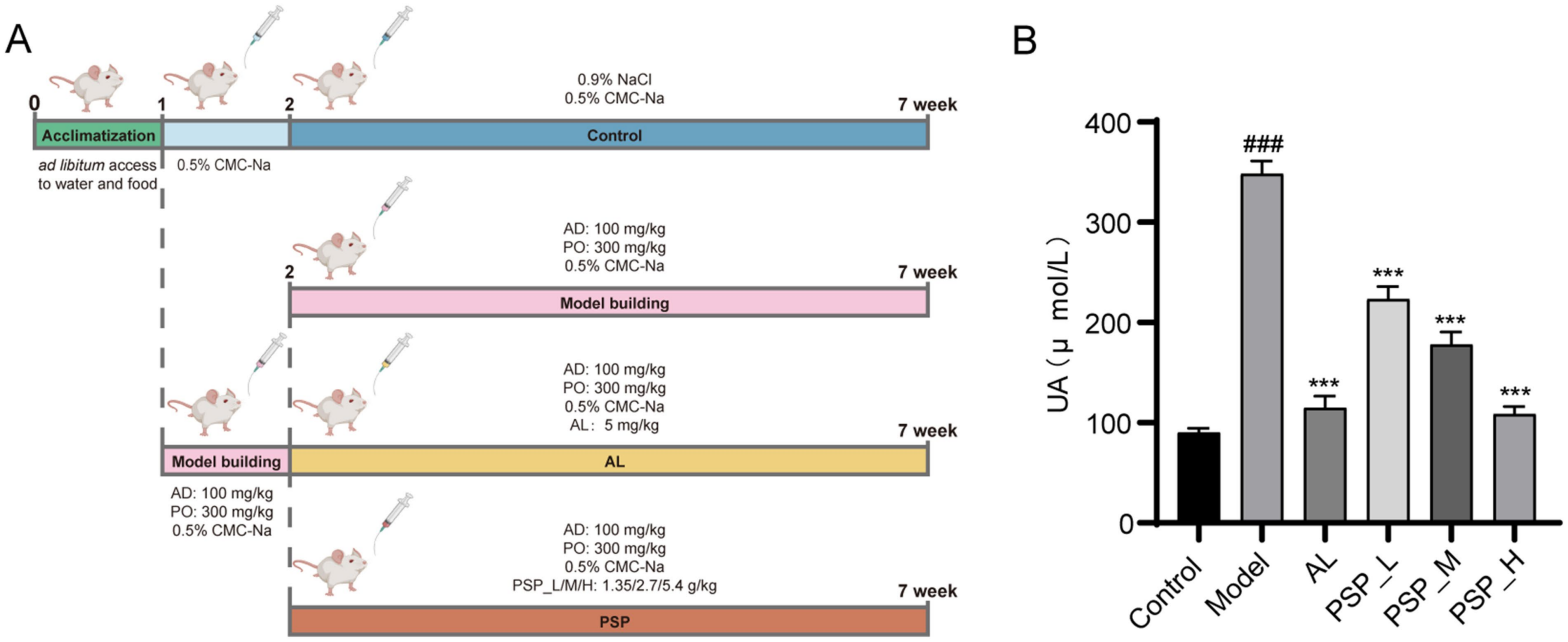
PSP improve HUA in rat models. Experimental design of PSP in the treatment of HUA in rats **(A)**. SUA levels **(B)**. ### represents *p* < 0.001 vs. the Control group; *** represents *p* < 0.001 vs. the Model group.

The establishment of the HUA rat model and the administration of PSP and AL are summarized in Figure 1A (Huijuan et al., 2017). The level of SUA increased significantly in the Model group after 1 week of model establishment. However, the level of SUA decreased after AL administration, indicating that the HUA rat model was successfully established (Liu et al., 2019). Compared with the Model group, SUA levels in HUA rats were significantly and dose-dependently reduced in PSP treatment groups(*p* < 0.001) (Figure 1B). These findings suggest that PSP can effectively alleviate HUA.

### 3.2 PSP alleviates renal damage in HUA rats

**Figure 2:**
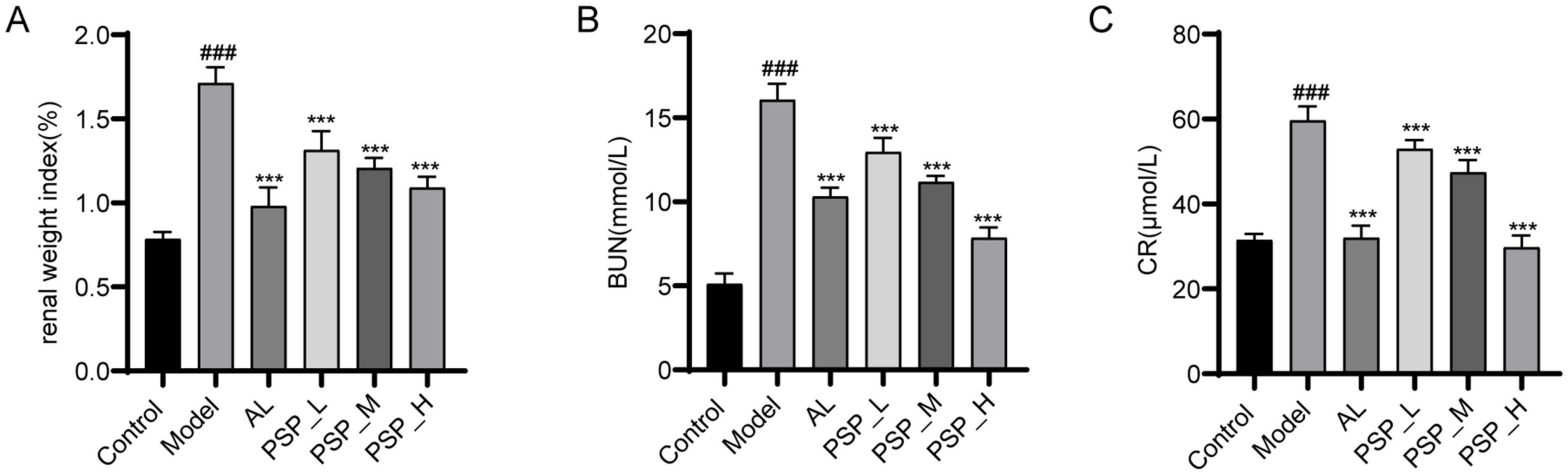
PSP treatment mitigated kidney damage in HUA rats. Kidney organ coefficient **(A)**. BUN levels **(B)**. CR levels **(C)**. ### represents *p* < 0.001 vs. the Control group; *** represent *p* < 0.001 respectively, vs. the Model group.

**Figure 3:**
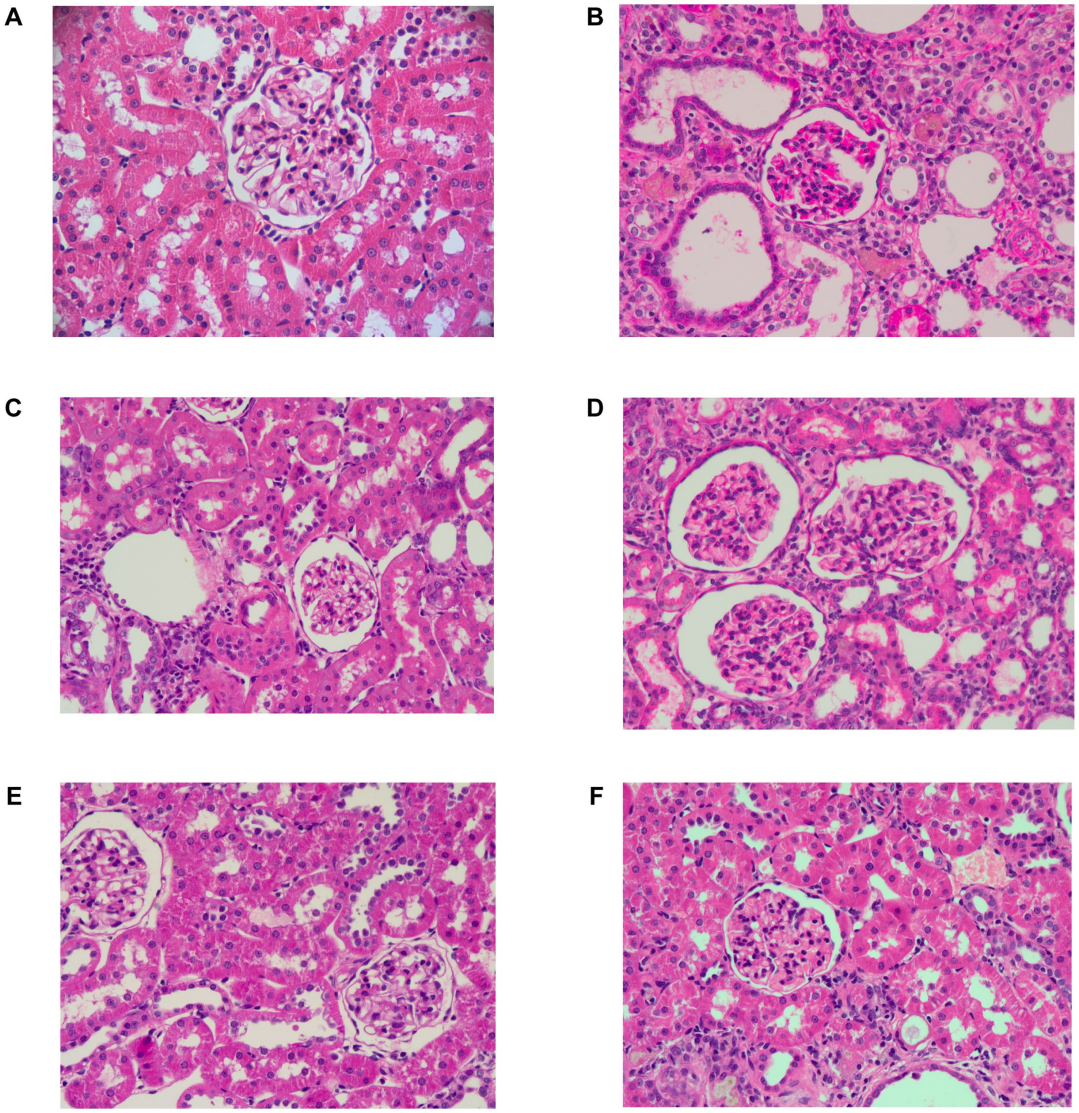
Representative H&E-stained pathological sections of the kidney (40×). Control group **(A)**; Model group **(B)**; AL group **(C)**; PSP_L group **(D)**; PSP_M group **(E)**; PSP_H group **(F)**.

HUA is often accompanied by an increase in renal burden (Yan et al., 2015). BUN and CR are commonly used to assess kidney excretory function. An increase in BUN usually reflects an increase in protein metabolism or a decrease in kidney excretory function, while CR more directly reflects the filtration capacity of the renal tubules. Thus, to verify the renal protective effect of PSP, the impact of PSP on renal function in HUA rats was studied using renal index, serum biochemical parameters CR and BUN, and histopathological examination. Compared with the control group, the model group exhibited a notable elevation in the renal index, BUN, and SCR levels. Furthermore, PSP treatment significantly and dose-dependently reduced the renal index, BUN, and SCR levels. AL, as a commonly used therapeutic drug, also reduced the levels of these indicators (*p* < 0.001) (Figure 2). HE staining results revealed that the kidney tissue morphology of the control group was normal, with a clear glomerular structure, uniform size, and intact tubular lumen. The kidney tissue of the Model group showed glomerular atrophy and tubular dilatation, which were significantly improved after PSP_L/M/H and 5 mg/kg/d AL treatment. The most significant improvement was observed in the PLP_H group (Figure 3). Collectively, these results indicate that PSP can effectively reduce renal damage in HUA rats.

### 3.3 PSP reduces serum XOD and ADA in HUA rats

**Figure 4:**
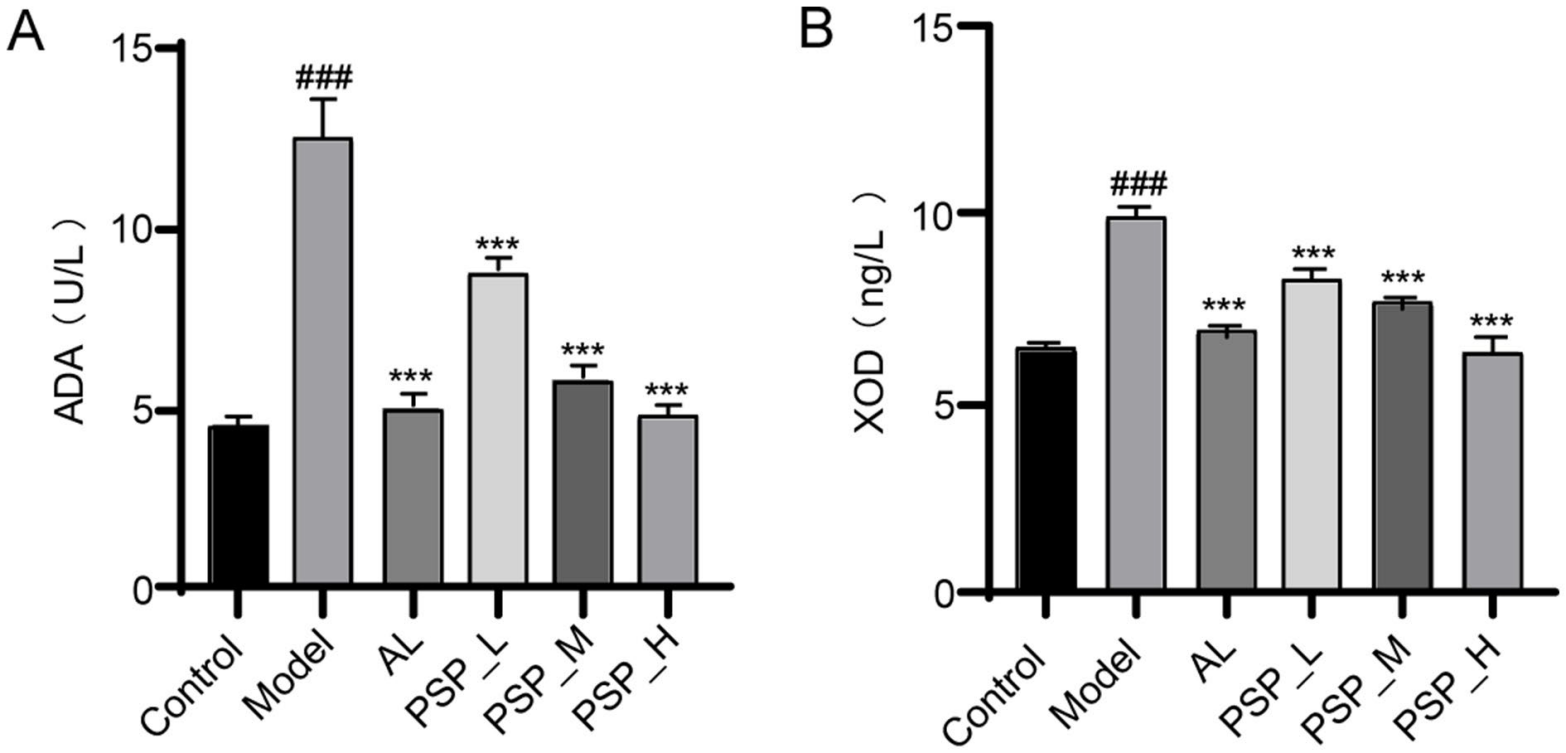
PSP reduces serum ADA and XOD in HUA rats in each group. Serum ADA **(A)**; serum XOD **(B)**; ### represents *p* < 0.001 vs. the Control group; *** represent *p* < 0.001 respectively, vs. the Model group.

ADA and XOD are the two rate-limiting enzymes in purine metabolism. The activities of serum ADA and XOD were markedly elevated in the model group compared with the control group (*p* < 0.001) (Figure 4). Compared with the model group, serum XOD and ADA activities were significantly and dose-dependently decreased in AL and all PSP groups. AL treatment also suppressed XOD and ADA levels. Taken together, these results suggest that the inhibition of XOD and ADA activities may be a mechanism for the UA-lowering function of PSP.

### 3.4 PSP alleviates metabolic disorders in HUA rats

**Figure 5:**
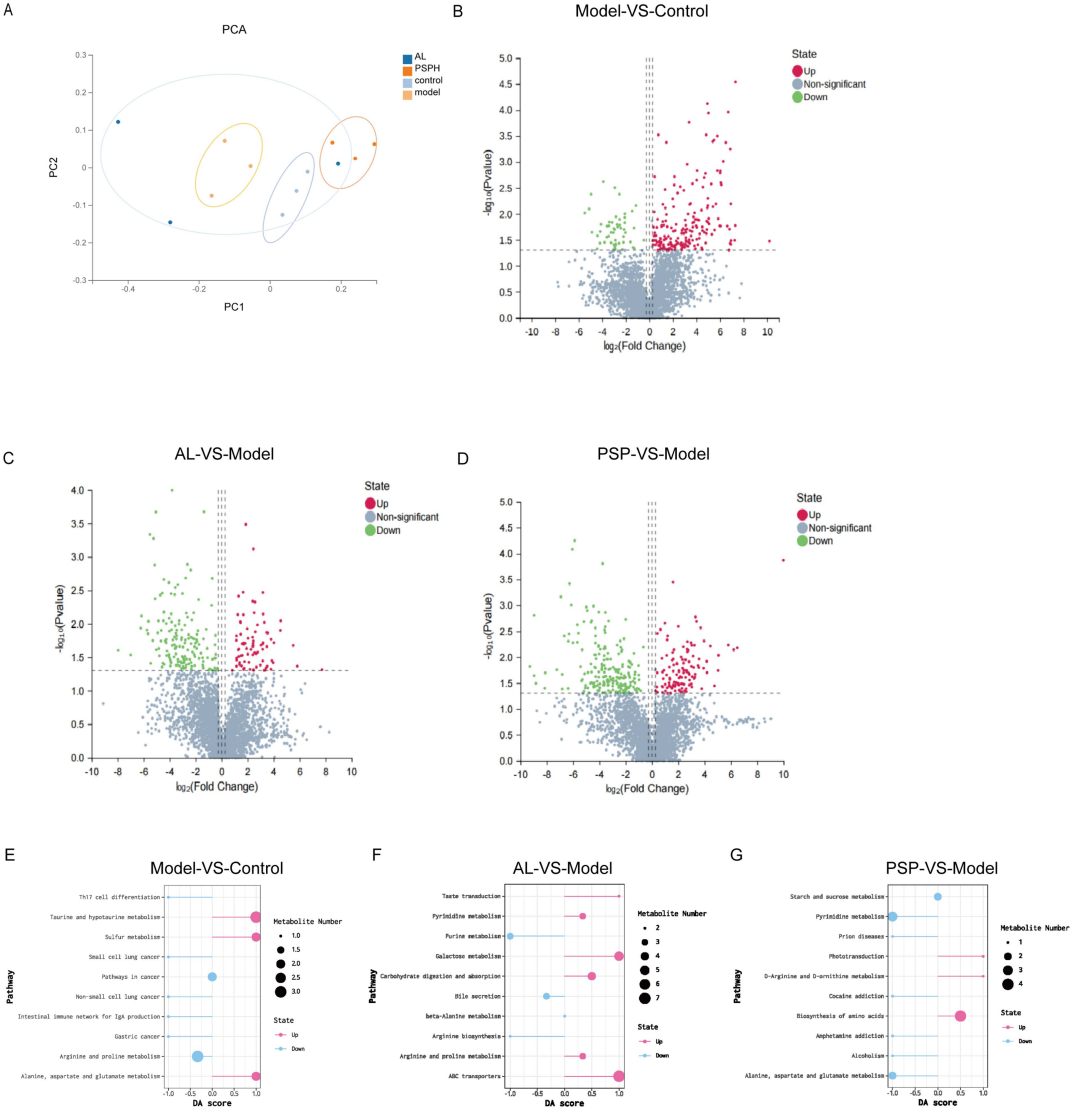
PSP improves the metabolic balance of HUA rats through intestinal flora. Principal component analysis score chart (PCA) **(A)**. VolcanoPlot of metabolite distribution **(B–D)**, Model vs. Control **(B)**. AL vs. Model **(C)**. PSP vs. Model **(D)**. Differential abundance score of metabolite distribution **(E–G)**, Model vs. Control **(E)**. AL vs. Model **(F)**. PSP vs. Model **(G)**.

Given the results of the previous biochemical and histopathological studies, we found that PSP can improve the biochemical indicators and pathological structure in HUA rats, and it has a dose-dependent effect. Therefore, we used the PSP_H group, which showed the best effect, for subsequent experiments. To comprehensively evaluate the mechanism of PSP on intestinal flora in HUA rats, a non-targeted metabonomic analysis of intestinal contents was performed, identifying 3,561 metabolites. Principal component analysis (PCA) showed that the PSP group was separated from the Model group, which was close to the Control group, indicating that PSP reversed the changes in intestinal flora metabolite composition in HUA rats (Figure 5A). Further analysis revealed that 201 differential metabolites were up-regulated and 53 were down-regulated in the Model group compared with the Control group (Figure 5B). However, compared with the Model group, the number of differential metabolites up-regulated after PSP and Al treatment was 123 and 82, and the number of differential metabolites down regulated was 210 and 160 (Figures 5C,D). These data further support the conclusion that PSP can improve the metabolic disorder of intestinal flora in HUA rats. Next, we analyzed the metabolic pathways and the abundance scores of metabolites to understand overall changes across the groups. It was found that the expression trend of differential metabolites in the alanine, aspartate, and glutamate metabolism pathway was up-regulated in the Model group compared with the Control group, and the number of metabolites in the pathway increased. However, the expression trend of differential metabolites was down-regulated in the arginine and proline metabolism pathway, and the number of metabolites in the pathway decreased. The expression trend of differential metabolites in the arginine and proline metabolism and pyrimidine metabolism pathway was up-regulated in the AL group compared with the Model group, and the number of metabolites in this pathway was increased. However, the expression trend of differential metabolites in the purine metabolism pathway was down-regulated, and the number of metabolites in this pathway was decreased. Additionally, the expression trend of differential metabolites in the pathway of biosynthesis of amino acids was up-regulated in the PSP_H group compared with the Mode group, and the number of metabolites in the pathway was increased. However, the expression trend of differential metabolites in the pyrite metabolism, alanine, aspartate, and glutamate metabolism pathway was down-regulated, and the number of metabolites in the pathway was decreased (Figures 5E–G). Overall, metabonomic analysis of intestinal contents provides evidence that PSP can improve HUA rats by regulating metabolites.

### 3.5 PSP restores the expression level of UA transporters in the kidneys of HUA rats and inhibits the inflammatory response

**Figure 6:**
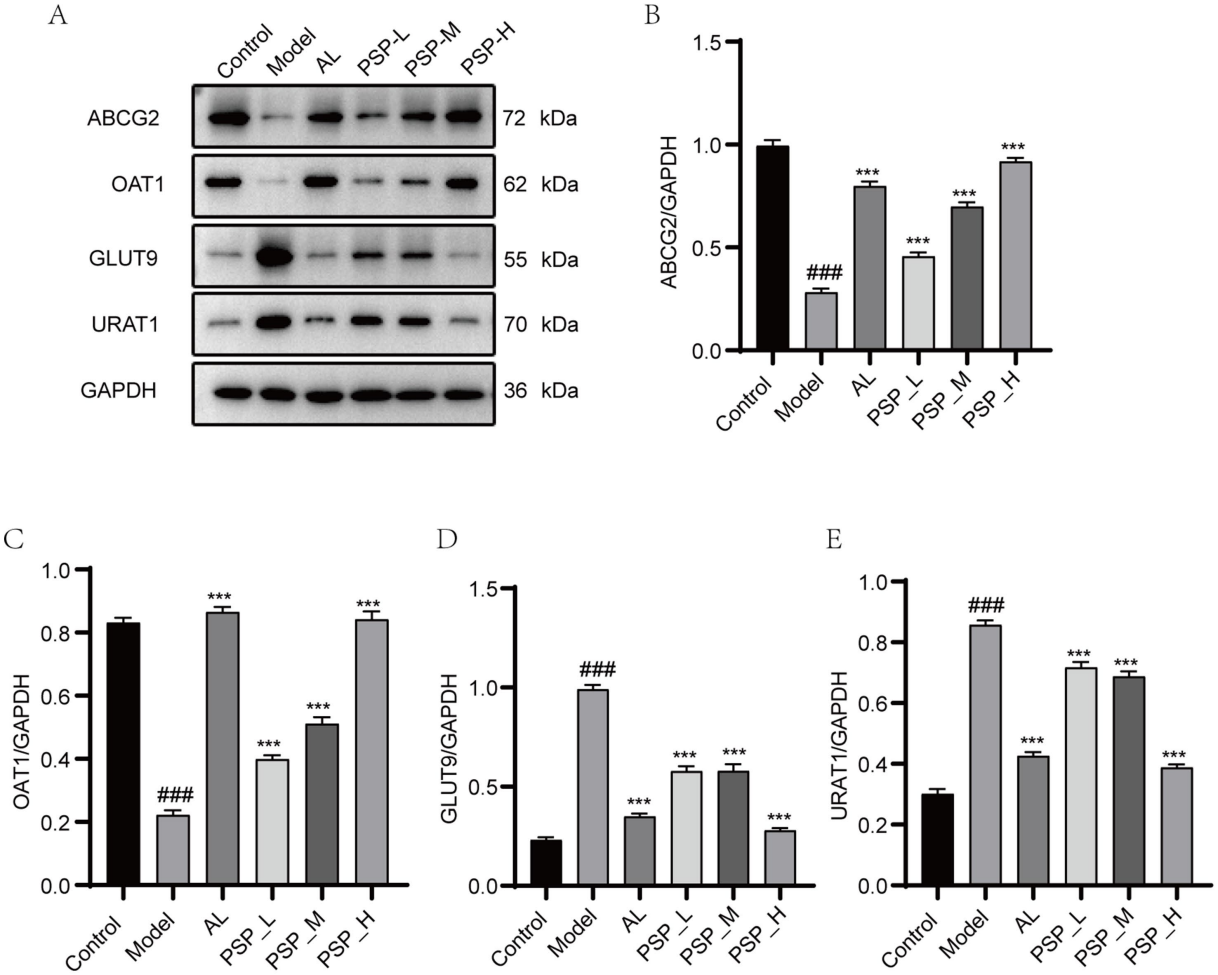
PSP reversed the aberrant expression of UA transporter proteins in the kidney of HUA rats. Western blots of ABCG2, OAT1, GLUT9 and URAT1 **(A)**. Quantification of ABCG2, OAT1, GLUT9 and URAT1 **(B–E)**. ### represents *p* < 0.001 vs. the Control group; *** represent *p* < 0.001 respectively, vs. the Model group.

UA reabsorption transporters (GLUT9 and URAT1) and UA excretion transporters (ABCG2 and OAT1) play an important role in the transport and excretion of UA in the kidney. We studied the expression of UA reabsorption and excretion transporters in the kidney at the protein level. Results showed that the expressions of URAT1 and GLUT9 were significantly increased, whereas the expressions of ABCG2 and OAT1 were significantly decreased in the Model group compared with the Control group. These results are consistent with previous studies (Zhang et al., 2023). Compared with the Model group, AL and all PSP treatments dose-dependently inhibited URAT1 and GLUT9, and significantly increased the gene and protein expression levels of ABCG2 and OAT1 in the kidney (Figure 6) (*p* < 0.001). Altogether, these findings indicate that PSP may enhance UA excretion by modulating UA transporter expression in the kidneys of hyperuricemic rats.

**Figure 7:**
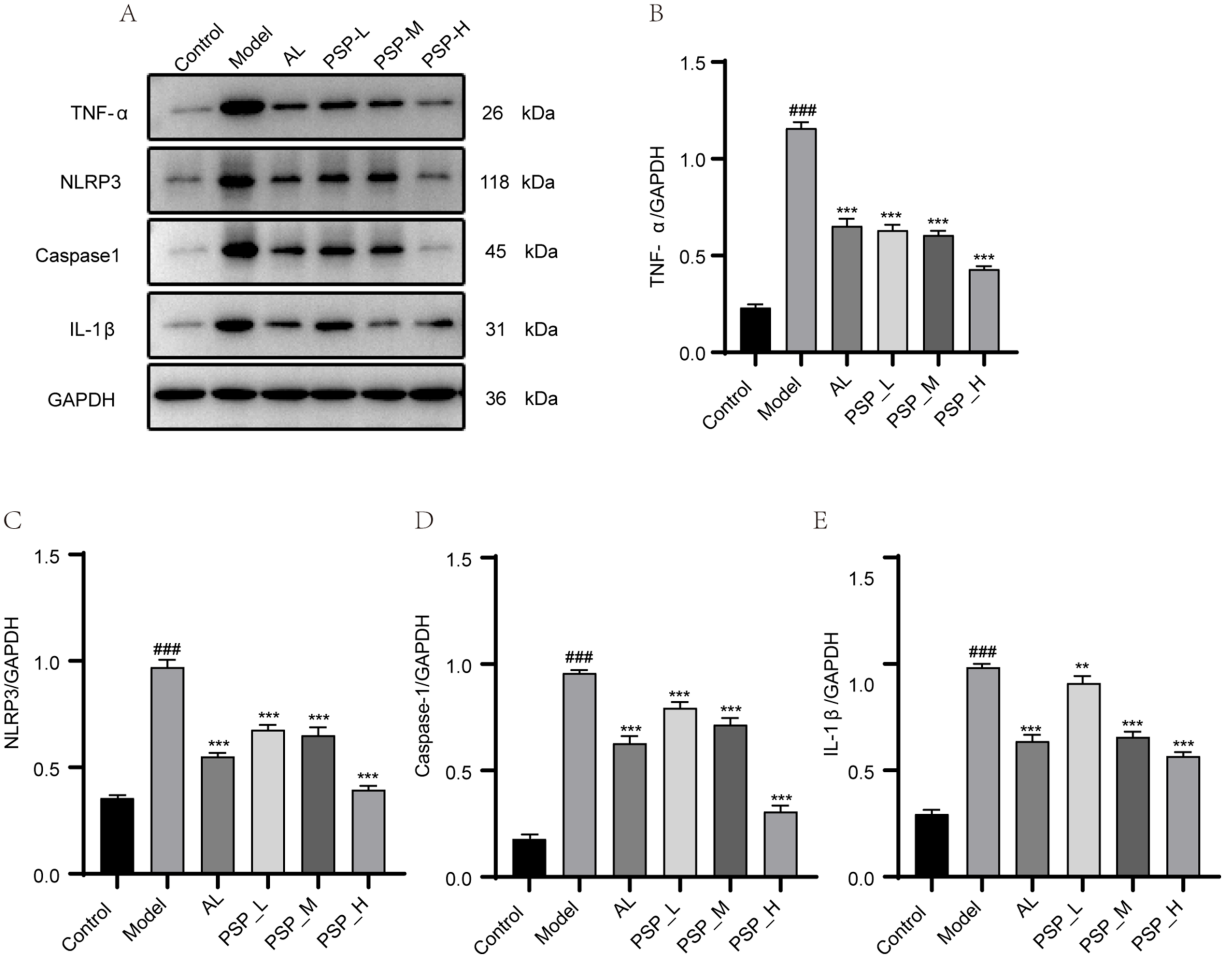
PSP can reverse renal inflammatory factors in HUA rats. Western blots of TNF-*α*, NLRP3, caspase-1 and IL-1β **(A)**. Quantification of TNF-α, NLRP3, caspase-1 and IL-1β **(B–E)**. ### represents *p* < 0.001 vs. the Control group; ** represent *p* < 0.01 respectively, vs. the model group.*** represent *p* < 0.001 respectively, vs. the Model group.

Elevated SUA concentrations result in the secretion of pro-inflammatory cytokines, consequently resulting in significant renal damage. UA activates NLRP3, which in turn activates caspase-1, ultimately mediating the expression of IL-1β and increasing the risk of nephritis. Therefore, we evaluated the effect of PSP on renal inflammation in HUA rats. The protein levels of TNF-α, NLRP3, caspase-1, and IL-1β in the kidney were significantly higher in the Model group than in normal rats. Compared with the Model group, AL and all PSP groups exhibited significantly decreased protein levels of these renal inflammatory factors (*p* < 0.001). Notably, the PSP_H group had a better inhibitory effect than the AL group (Figure 7). Together, these results demonstrate that PSP can inhibit inflammation by inhibiting the NLRP3/caspase-1 inflammatory pathway in the kidney of HUA rats, thereby contributing to the amelioration of renal injury.

### 3.6 PSP alleviates the injury of the intestinal mucosal barrier in HUA rats

**Figure 8:**
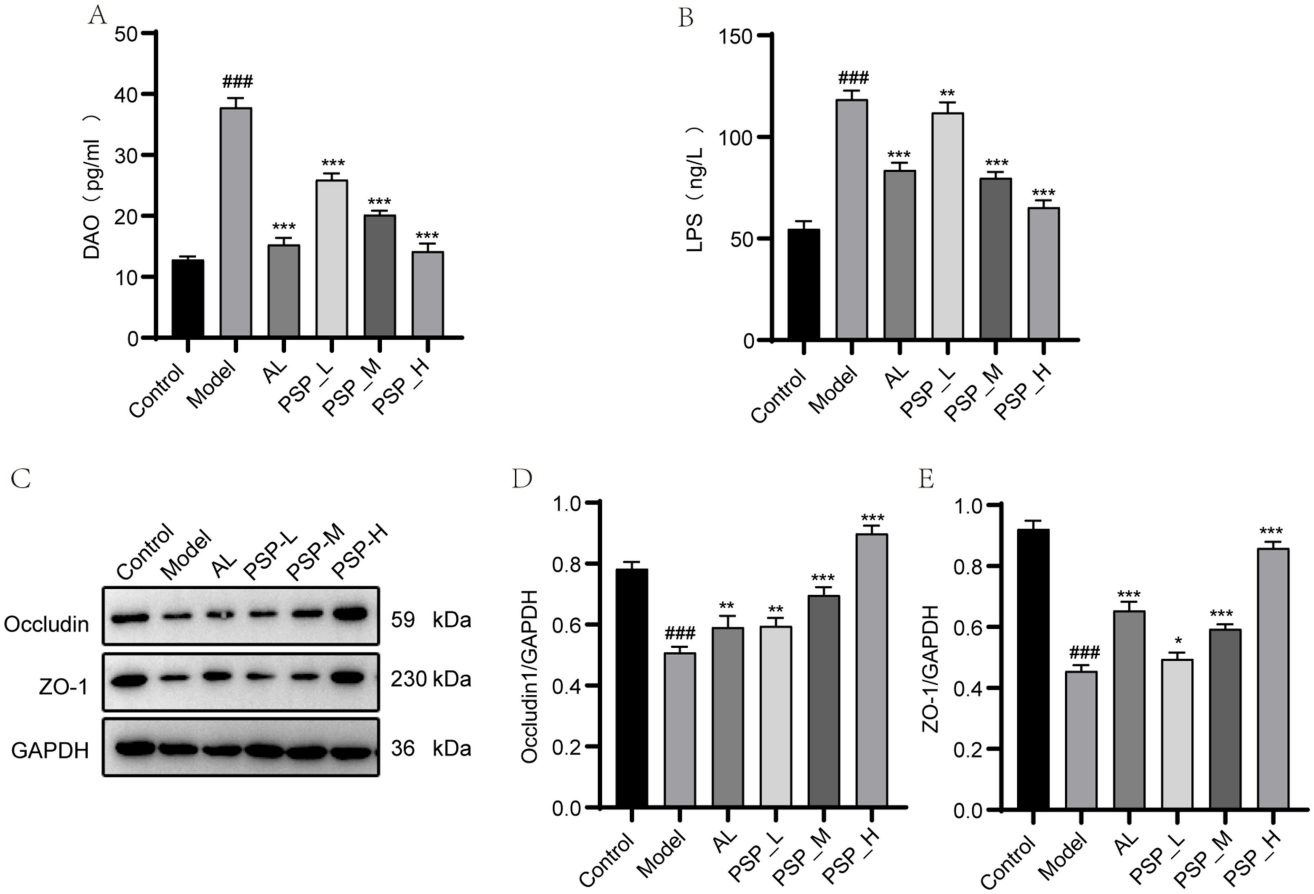
Comparison of intestinal mucosal barrier indicators and intestinal tight junction protein levels in each group of rats. Serum DAO **(A)**; serum LPS **(B)**; intestinal occludin of western blots and quantification **(C,E)**; intestinal ZO-1 of western blots and quantification **(D,E)**. ### represents *p* < 0.001 vs. the Control group; * represent *p* < 0.05 respectively, vs. the Model group; ** represent *p* < 0.01 respectively, vs. the model group; *** represent *p* < 0.001 respectively, vs. the Model group.

DAO and LPS are serum biomarkers of increased intestinal barrier permeability. The results showed that serum DAO and LPS levels were significantly higher in the Model group than in the Control group (*p* < 0.01, Figures 8A,B). Meanwhile, the serum level of LPS was significantly reduced in AL and all PSP groups compared with the Model group (*p* < 0.001).

In addition, the intestinal tight junction proteins occludin and ZO-1 play a key role in maintaining intestinal integrity and internal environmental homeostasis. The expression levels of occludin and ZO-1 proteins were lower in the Model group than in the Control group (*p* < 0.05, Figures 8C–E). However, this effect was reversed by AL and PSP treatments, with the most significant effect observed in the PSP_H group (*p* < 0.05). Collectively, these results suggest that PSP can promote the repair of the intestinal barrier in HUA rats.

### 3.7 PSP improves intestinal flora dysbacteriosis in HUA rats

**Figure 9:**
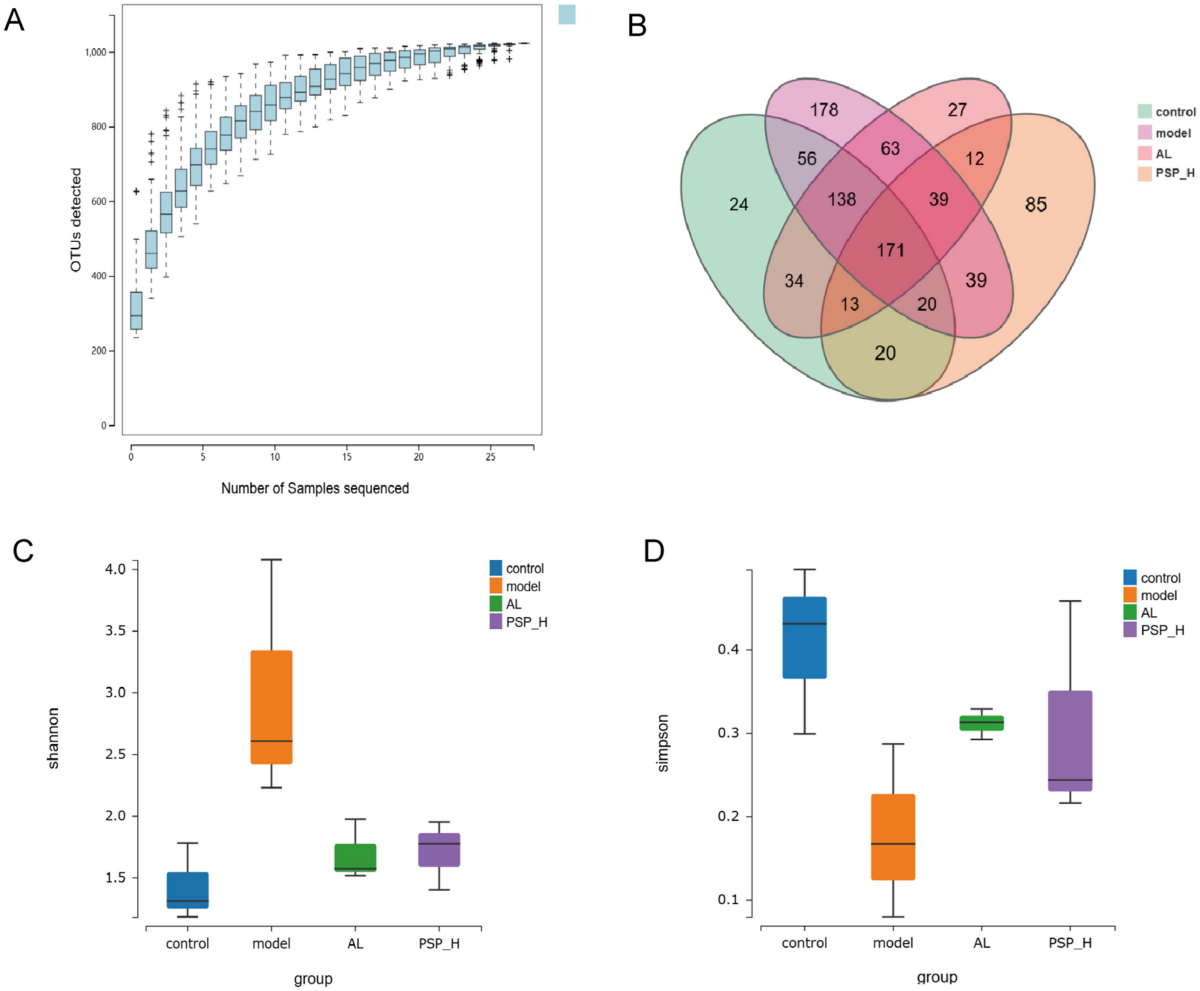
The effect of PSP on intestinal flora diversity of HUA rats. Species accumulation curve **(A)**; Venn diagram **(B)**; Shannon’s index **(C)**; Simpson’s index **(D)**.

**Figure 10:**
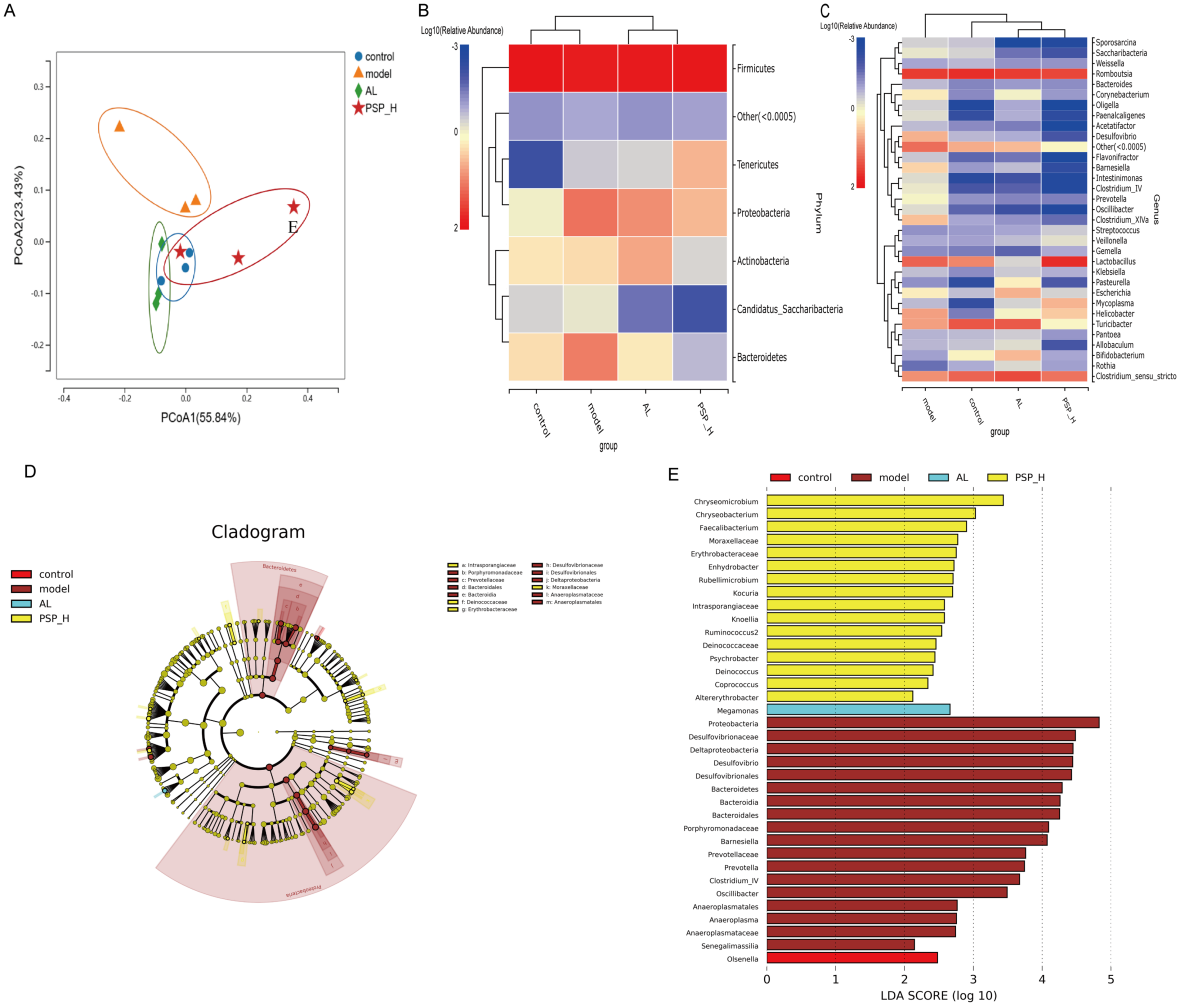
The impact of PSP on the similarity of intestinal flora composition in Hua rats. Principal coordinates analysis **(A)**. Heat map of species composition at the phylum level **(B)**; Heat map of species composition at the genus level **(C)**; linear discriminant analysis effect size (LEfSe) analysis **(D,E)**.

It is well-established that two-thirds of UA excretion occurs through the kidney and one-third through the intestines, and UA is metabolized by the intestinal microbiome (Lv et al., 2020). Therefore, 16S ribosomal RNA (rRNA) sequencing was performed to further determine whether PSP protects the intestine and kidney through intestinal flora, thereby reducing HUA. Based on the preliminary results, we selected the PSP_H group with the best efficacy for the study of intestinal microbiota. Species accumulation curves are shown in Figure 9A. The curves rise sharply before leveling off as the number of samples increases, indicating that the sample size was reasonable for data analysis. The Venn diagram revealed that 171 Operational Taxonomic Units (OTUs) were shared among the four groups, as well as the number of OTUs unique to each group (Figure 9B). Alpha diversity analysis revealed the diversity of each group of communities. The Shannon index was higher in the Model group than in the Control group, which was reversed in the AL and PSP_H groups. The Simpson index was negatively correlated with species diversity. The Simpson index was lower in the Model group than in the Control group, indicating that the diversity of intestinal flora in rats increases under the effect of high HUA, which was also reversed in the AL group and PSP_H groups (Figures 9C,D). These results showed that the intestinal microbial richness in the Model group increased under the action of HUA, and AL and PSP treatments restored the diversity of intestinal microflora. Moreover, β-diversity analysis of the similarity of gut microbial composition between communities was performed. Principal Coordinate Analysis (PCoA) showed that the Control and Model groups were divided into different clusters, which was reversed by PSP and AL (Figure 10A), indicating that PSP and AL restored the composition of intestinal flora. The distance between the Control group and the AL and PSP groups was close, and the difference in community composition was small. Taken together, these results implied that PSP had a positive regulatory effect on intestinal microbial structure.

To gain a deeper insight into the particular alterations within microbial communities, we examined the relative abundance of predominant bacterial profiles at both the phylum and genus levels. It was found that *Firmicutes* was the dominant phylum. The composition and structure of intestinal microbiota changed significantly and the ratio of *Firmicutes*/*Bacteroidetes* was decreased in the Model group compared with the Control group. However, the PSP_H treatment reversed this phenomenon. The relative abundance of *Bacteroidetes* and *Proteobacteria* was decreased, the relative abundance of *Firmicutes* was increased, and the ratio of *Firmicutes*/*Bacteroidetes* increased in PSP groups compared with the Model group. These results indicate that PSP can restore the intestinal flora homeostasis in HUA rats. Meanwhile, *Romboutsia* was the most abundant intestinal microbiota in the four groups. *Romboutsia*, *Clostridium_sensu_stricto*, and *Turicibacter* were decreased and *Lactobacillus* was increased in the Model group compared with the Control group. Moreover, *Romboutsia* and *Clostridiu_sensu_stricto* were improved in varying degrees in the AL and all PSP groups compared with the Model group (Figures 10B,C). The relative abundance of *Lactobacillus* was increased in PSP_H compared with the AL group, indicating that the two drugs may have different regulatory mechanisms. Linear discriminant analysis Effect Size (LEfSe) analysis was performed to explore the variation in characteristic differential bacteria in rats (Figures 10D,E). Eighteen types of bacteria, including *Proteobacteria* and *Desulfovibrionaceae*, were enriched in the Model group, whereas 16 types of bacteria, including *Chryseomicrobium* and *Chryseobacterium*, were enriched in PSP groups. *Megamona*s was enriched in the AL group. Overall, these findings indicate that the gut microbiota in PSP and AL groups exhibit distinct regulatory effects on UA levels.

## 4 Discussion

Establishing reasonable and effective animal models is essential for screening anti-UA drugs. Currently, pharmacological interventions are predominantly utilized to induce HUA models in rodents. This is achieved by either increasing the intake of UA inhibiting uricase activity, reducing UA excretion, or employing a combination of these approaches (Daignan-Fornier and Pinson, 2019; Mehmood et al., 2020). Based on the literature and preliminary experiments, the present study utilized a combination of potassium oxonate (a competitive uricase inhibitor) and yeast powder and adenine (urate precursors) to establish a rat model of HUA that closely mimics human conditions (Huijuan, et al., 2017). As anticipated, SUA levels were higher in the model rats than in normal rats, and this difference was statistically significant. The increase underscores the efficacy of the combination therapy and the simple reproducibility of the operation. Both PSP and AL improved this hyperuricemic state, indicating that PSP is effective in stabilizing and normalizing UA levels, making it a potential agent for managing HUA.

Elevated UA levels are known to induce nephrotoxicity and can increase the risk of renal failure (Gherghina et al., 2022). SCR and BUN, excreted by the kidneys, are vital indicators of kidney function, with elevated levels serving as hallmarks of kidney failure. Our model exhibited several characteristics and pathological conditions akin to clinical manifestations, including SCR and BUN concentrations and impaired renal function. After PSP administration, BUN and SCR levels decreased compared with the Model group, indicating that PSP alleviated the renal insufficiency induced by high UA. AL, as a positive control, demonstrated a lesser impact on BUN and SCR recovery than the PSP_H group. Renal histopathology also corroborated these findings. The improvement in the AL group was not significant, indicating poor recovery of renal tissue. In contrast, the PSP_H group showed a remarkable improvement, successfully repairing renal tissue damage. These findings indicate that PSP may lower UA levels and improve kidney function by mitigating the impact on the renal tubules and glomeruli.

Higher UA production may result in HUA. ADA and XOD, key enzymes in the end stage of purine catabolism, directly regulate UA production (Keenan, 2020; Kutryb-Zajac et al., 2020; Serrano et al., 2020). Enzyme activity abnormalities are considered a significant factor in UA overproduction, which further leads to HUA. The present research revealed that XOD and ADA activities in hyperuricemic rats increased following the administration of the modeling drug, leading to heightened purine metabolism and elevated UA levels. Both PSP and AL treatment reduced the levels of the two enzymes, with PSP_H exhibiting comparable effects to AL. These findings indicate that PSP can effectively inhibit XOD and ADA.

Reduced UA excretion is another pathogenic mechanism. Therefore, promoting UA excretion is considered an essential strategy for the prevention and treatment of HUA. The kidney facilitates the removal of UA load primarily through the synergy of a complex series of urate transport proteins, making it a potential target for treating HUA (Sun et al., 2021). URAT1 and GLUT9, major transporters involved in UA reabsorption, are localized on the apical surface, basolateral membrane, and apical membrane of renal tubular cells (Adomako and Moe, 2023; Chen et al., 2021a). ABCG2 and OAT1 are located on the apical brush border and the basolateral membrane. These proteins regulate the excretion of UA from the blood to the lumen of proximal tubules. Previous studies have shown that mice with URAT1, GLUT9, ABCG2, or OAT1 deficiency in the kidney are prone to HUA (Nigam et al., 2015). This suggests that the UA transporter is a key target for the prevention and treatment of HUA. The current study found that PSP reduced URAT1 and GLUT9 expression but increased ABCG2 and OAT1 expressions in the kidney of HUA model rats, similar to AL treatment. However, the impact of PSP was greater than that of AL, indicating that PSP might be more effective in combating HUA by enhancing the kidney’s ability to excrete UA.

Recent research has focused on the association between HUA and intestinal microecology. The intestinal barrier is integral to the preservation of intestinal homeostasis. Similarly, tight proteins in the intestinal epithelial maintain intestinal mucosal barrier function. ZO-1 and occludin are recognized as the key proteins in this process (Kuo et al., 2022). Increased intestinal permeability is a sign of an impaired mucosal barrier. DAO and LPS are the metabolites of gram-negative bacteria (Zheng et al., 2021). Dysbacteriosis has the potential to adversely affect the intestinal barrier and its integrity, thereby facilitating the invasion of intestinal pathogens, LPS, and other toxins into the kidneys via the bloodstream. This process may exacerbate inflammation, injury, and dysfunction in the kidneys, ultimately resulting in inadequate UA excretion. Our research indicated that PSP enhanced intestinal function by modulating DAO, LPS, and tight junction proteins. It effectively prevented the translocation of bacteria and LPS into the intestine while alleviating pathological changes. PSP also reversed HUA-induced intestinal environmental homeostasis disturbance and maintained the integrity of the intestinal mechanical barrier. These findings establish a strong foundation for regulating the intestinal microecology of HUA.

PSP-induced changes in gut microflora may help alleviate HUA-related kidney injury. Recent studies have unveiled the possible involvement of intestinal microflora in metabolic diseases (Fan and Pedersen, 2021). Numerous studies have demonstrated a strong connection between intestinal microflora and HUA (Yu et al., 2018). An elevated SUA level along with a significant release of UA into the intestinal cavity can influence the physiological environment of the gut flora, altering both its structure and abundance (Hobby et al., 2019). Dysbiosis exacerbates the progression of hyperuricemia through metabolic-inflammation pathways: dysbiosis leads to a reduction in beneficial bacteria and an increase in pathogenic bacteria, the former weakening the inhibition of hepatic purine metabolic enzymes (ADA/XOD), while the latter activates the TLR4/NF-κB pathway and triggers the NLRP3 inflammasome, promoting IL-1β release and imbalances in renal tubular uric acid transport. PSP breaks the microbiota-metabolism-inflammation vicious cycle by adjusting the composition of the gut microbiota, which simultaneously repairs gut barrier function, inhibits inflammatory cascades, and regulates renal uric acid transport proteins. In this study, the relative abundance of *Bacteroidetes* and *Firmicutes* decreased in hyperuricemic rats. PSP altered the structure of the gut microbiota. Compared to the Model group, it first regulated the diversity of the gut microbiota; secondly, it corrected the dysbiosis by reducing *Bacteroidetes* and *Proteobacteria* while increasing *Firmicutes* and *Tenericutes*. The F/B ratio is associated with gut microbiota disorder and systemic inflammation. After PSP intervention, at the phylum level, *Firmicutes* significantly increased, while the relative abundances of *Bacteroidetes* and *Proteobacteria* rose; at the genus level, *Romboutsia* and *Clostridium_sensu_stricto* increased, while *Bacteroides* decreased. The increased abundance of *Firmicutes* directly promotes the synthesis of SCFAs (such as butyrate), which inhibits histone deacetylase (HDAC) by activating the GPR43 receptor, downregulating LPS and TNF-α expression. These suggest that PSP can alleviate HUA, inflammation, and kidney and intestinal injury by remodeling intestinal flora (Li et al., 2015). However, PSP plays a protective role by influencing the intestinal flora. This suggests that adjusting the composition of the intestinal flora could enhance HUA and its associated complications (He et al., 2023; Shi et al., 2017).

Inflammation is a clinicopathological feature of HUA triggered by the accumulation of supersaturated UA that can lead to kidney damage (Jung et al., 2020). In addition, enhanced intestinal-derived LPS translocation can activate inflammatory pathways, worsening inflammation and leading to kidney damage. Given the profound effects of inflammation and tissue injury in various diseases, there is a need for UA-lowering drugs that offer both anti-inflammatory and kidney-protecting benefits. TNF-α is positioned upstream in the inflammatory cascade response and stimulates the production of IL-1β, which can be markedly increased due to UA stimulation (Hira and Sajeli Begum, 2021). NLRP3 is an inflammation danger signal directly activated by UA. The activation of inflammatory vesicles significantly enhances the release of caspase-1 and IL-1β (Xu et al., 2021). IL-1β, a key pro-inflammatory mediator, is crucial in gout development (Han et al., 2020). UA and LPS elevated inflammatory factors in Model group rats, and high-dose PSP was more effective than AL in reducing pro-inflammatory factors. These findings indicate that PSP can reduce and manage inflammatory signals, thus preventing renal damage.

## 5 Conclusion

In summary, this study demonstrates that PSP effectively improves HUA in rats by restoring kidney and intestinal functions. PSP can also improve the protein expression of renal transporters by inhibiting the activation of the inflammatory signaling pathway, and repair the intestinal mucosal barrier to inhibit the translocation of lipopolysaccharides. In addition, PSP regulates intestinal microbiota and metabolic imbalances in HUA rats. This study has unraveled how PSP reduces UA in HUA and examined its mechanisms in the kidney and intestine. Based on the natural properties of PSP and its lack of liver and kidney toxicity, it can be considered as a prebiotic for the preparation of personalized dietary supplements. These findings provide novel insights into HUA treatment.

## Data Availability

The original contributions presented in the study are publicly available. This data can be found here: https://ngdc.cncb.ac.cn/, accession number PRJCA037431.
